# 2768. In Vitro Antibiotic Susceptibility Pattern of Non-*diphtheriae Corynebacterium* Isolates 2012-2023, reference lab experience

**DOI:** 10.1093/ofid/ofad500.2379

**Published:** 2023-11-27

**Authors:** Omar M Abu Saleh, Said El Zein, Christina G Rivera (O'Connor), Ryan W W Stevens, Audrey N Schuetz, Madiha Fida

**Affiliations:** Mayo Clinic Rochester, Rochester, Minnesota; Mayo Clinic, Rochester, Minnesota; Mayo Clinic, Rochester, Minnesota; Mayo Clinic, Rochester, Minnesota; Mayo Clinic, Rochester, Minnesota; Mayo Clinic, Rochester, Minnesota

## Abstract

**Background:**

Non-*diphtheriae Corynebacterium* spp. are important human pathogens responsible for a broad spectrum of clinical syndromes. Data on species-specific susceptibility patterns is limited. In this study we aim to study these susceptibility patterns over time and summarize the species-specific resistance profile that could be used by clinicians to guide therapeutic decisions.

**Methods:**

We conducted a retrospective review of all the Non-*diphtheriae Corynebacterium* isolates that were submitted for antimicrobial susceptibility testing (AST) between January-2012 and February of 2023 to our reference lab.

**Results:**

A total of 1925 *Corynebacterium* isolates were submitted for susceptibility testing over the study period. The most commonly identified species was *C. striatum* (35.6%) followed by *C. amycolatum* (24.4%). Figure-1 outlines the species distribution. Temporal analysis showed decreasing susceptibility rates over the study period from 47.5% to 20.6% for penicillin & from 50% to 7.6% for clindamycin.

Linezolid and vancomycin were universally active against all species (Table-1). Daptomycin non-susceptibility was reported in 25% of C. jeikeium isolates (MIC=2). High level resistance to daptomycin with MIC >256 were noted in seven *C. striatum* and single *C. amycolatum* isolate. Doxycycline has the highest susceptibility rates among the tested oral agents after linezolid for the non-striatum species.

Significant variation in species-specific susceptibility pattern was noted and summarized in Table 1. *C. striatum* was associated with concerning resistance rates to beta-lactams, tetracyclines, trimethoprim-sulfamethoxazole & fluoroquinolones with limited reliable oral treatment options beside linezolid. *C. kroppenstedtii* (associated with granulomatous mastitis) showed high susceptibility rate to tetracyclines and trimethoprim-sulfamethoxazole despite prevalent penicillin resistance.

**Figure:**
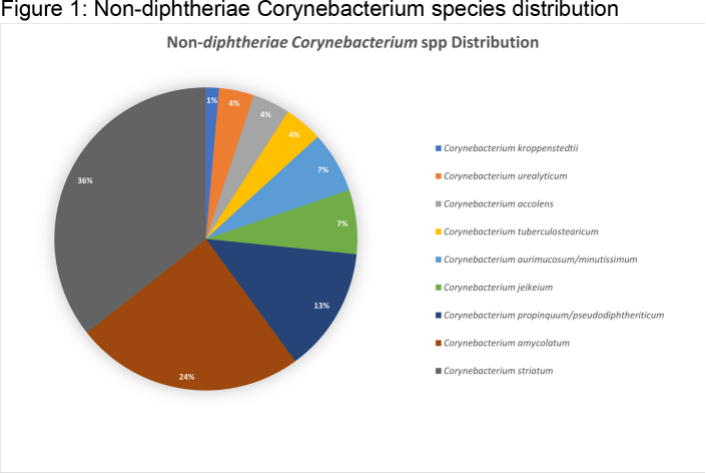

**Figure:**
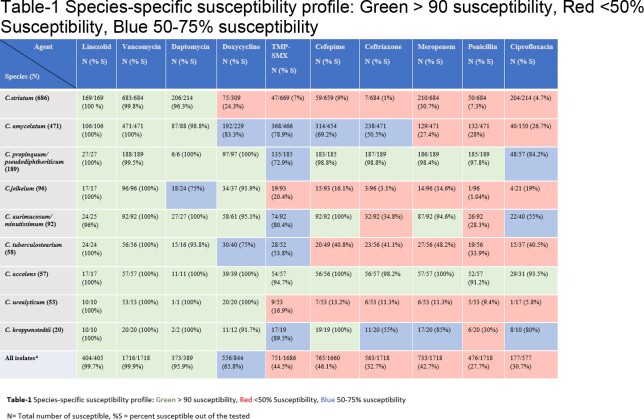

**Conclusion:**

Non-*diphtheriae Corynebacterium* spp are commonly isolated from clinical specimens and are associated with various clinical syndromes. Significant variation in resistance profile exists between different species. Prevalent resistance among the most common species such as *C. Striatum* is associated with therapeutic challenges.

**Disclosures:**

**Christina G. Rivera (O'Connor), Pharm.D**, Gilead Sciences: Advisor/Consultant|Gilead Sciences: Board Member|Gilead Sciences: Grant/Research Support|Gilead Sciences: Honoraria **Audrey N. Schuetz, MD**, Merck: Advisor/Consultant

